# Lactoferrin Mediates Enhanced Osteogenesis of Adipose-Derived Stem Cells: Innovative Molecular and Cellular Therapy for Bone Repair

**DOI:** 10.3390/ijms24021749

**Published:** 2023-01-16

**Authors:** Yiqiang Chang, Ansong Ping, Chunyu Chang, Volker M. Betz, Lin Cai, Bin Ren

**Affiliations:** 1 Department of Orthopedics, Zhongnan Hospital of Wuhan University, Wuhan 430070, China; 2 College of Chemistry and Molecular Sciences, Engineering Research Center of Natural Polymer-based Medical Materials in Hubei Province and Laboratory of Biomedical Polymers of Ministry of Education, Wuhan University, Wuhan 430072, China; 3 Department of Orthopedics and Trauma Surgery, Musculoskeletal University Center Munich (MUM), University Hospital LMU Munich, 81377 Munich, Germany

**Keywords:** lactoferrin, BMP-2, bone tissue engineering, adipose derived stem cells, molecular therapy, cell therapy

## Abstract

A prospective source of stem cells for bone tissue engineering is adipose-derived stem cells (ADSCs), and BMP-2 has been proven to be highly effective in promoting the osteogenic differentiation of stem cells. Rarely has research been conducted on the impact of lactoferrin (LF) on ADSCs’ osteogenic differentiation. As such, in this study, we examined the effects of LF and BMP-2 to assess the ability of LF to stimulate ADSCs’ osteogenic differentiation. The osteogenic medium was supplemented with the LF at the following concentrations to culture ADSCs: 0, 10, 20, 50, 100, and 500 μg/mL. The Cell Counting Kit-8 (CCK-8) assay was used to measure the proliferation of ADSCs. Calcium deposition, alkaline phosphatase (ALP) staining, real-time polymerase chain reaction (RT-PCR), and an ALP activity assay were used to establish osteogenic differentiation. RNA sequencing analysis was carried out to investigate the mechanism of LF boosting the osteogenic development of ADSCs. In the concentration range of 0–100 μg/mL, LF concentration-dependently increased the proliferative vitality and osteogenic differentiation of ADSCs. At a dose of 500 μg/mL, LF sped up and enhanced differentiation, but inhibited ADSCs from proliferating. LF (100 and 500 μg/mL) produced more substantial osteoinductive effects than BMP-2. The PI3 kinase/AKT (PI3K/AKT) and IGF-R1 signaling pathways were significantly activated in LF-treated ADSCs. The in vitro study results showed that LF could effectively promote osteogenic differentiation of ADSCs by activating the PI3K/AKT and IGF-R1 pathways. In our in vitro investigation, an LF concentration of 100 μg/mL was optimal for osteoinduction and proliferation. Our study suggests that LF is an attractive alternative to BMP-2 in bone tissue engineering. As a bioactive molecule capable of inducing adipose stem cells to form osteoblasts, LF is expected to be clinically used in combination with biomaterials as an innovative molecular and cellular therapy to promote bone repair.

## 1. Introduction

Patients with bone deformities brought on by trauma, infection, tumors, or other illnesses experience functional, aesthetic, and psychological issues [1]. As a promising therapeutic alternative for bone repair and regeneration, pluripotent stem cell-based tissue engineering techniques for regenerative bone repair have transformed current therapy paradigms [2]. Bone tissue effectively produced from culture-expanded stem/progenitor cells through in vivo transplantation has received considerable interest [3]. One of the most pressing issues in regenerative medicine is how to benefit from the therapeutic potential of stem cells for use in bone repair and regeneration.

Mesenchymal stem cells were originally thought to primarily originate in the bone marrow. Donor site pain and inadequate cell yield are just two problems that might arise from using bone-marrow-derived stem cells (BMSCs) in a therapeutic setting [4], which have led to continuous efforts to find alternative sources to mesenchymal stem cells. The adipose-derived stem cell (ADSC) is a type of pluripotent progenitor cell that is capable of self-renewal and cell type differentiation [5,6,7]. Mounting evidence show that ADSCs have considerable potential as a regenerative therapeutic tool due to their ease of separation, readily available sources, lack of immunogenicity, and low donor morbidity [8]. Several biomedical strategies have been suggested to increase the osteogenic capacity of ADSCs to use their therapeutic potential for bone repair. These strategies include genetically altering stem cells [5,9,10,11], using scaffolds with osteogenic activity [5,12,13,14], and using bioactive molecules [3,15,16,17], etc. Interestingly, stem cell differentiation using bioactive molecules appears to be a novel and effective method to encourage regeneration. This technology has great potential and may get beyond ethical concerns and the restrictions of genetic modification.

Members of the superfamily of transforming growth factor-beta (TGF-β) are known as bone morphogenetic proteins (BMPs) [18]. They are categorized into numerous groups, including BMP (−2 and −4), BMP (−5, −6, −7, and −8), and BMP (−9, −10, and −15) [19]. Different BMP subtypes are crucial for the osteogenic development of other mesenchymal stem cells. In particular, the United States of America, Europe, and Australia have authorized BMP-2 and BMP-7 in clinical settings [4]. In tissue engineering, growth factor delivery-based therapeutic strategies have recently become more prevalent. The high cost burden, huge molecular weight at physiological levels, inability to sustain long-term concentrations, and toxicity at high doses are some of the constraints that have hindered the success of clinical trials with rh-BMP-2.

Lactoferrin (LF) is a member of the transferrin family of iron-binding glycoproteins. LF can be found in exocrine fluids such as milk, saliva, and other biological secretions produced by mammals [20]. It has been reported that LF is a decisive anabolic bone growth factor and may work as an effector molecule for remodeling the bone [21]. Orally administered LF significantly halted estrogen deficiency induced bone loss and enhanced bone microarchitecture in ovariectomized rats [22], which generated substantial interest in bone regeneration as a functional food ingredient addition, or a potential bioactive protein. However, the oral administration of LF often requires large doses and may induce off-target side effects, including undesirable heterotopic ossification.

Following this, the results of several in vitro experiments showed that the local distribution of LF strongly increases the proliferation and differentiation of osteoblasts from rats to humans [23,24,25], which raised serious doubts about LF’s potential as a growth factor in the bone tissue engineering field. The impact of LF on stem cell osteogenic differentiation has, however, barely been studied. Furthermore, nothing is known about the molecular pathways that underlie the influence of LF on osteogenic differentiation.

The purpose of our study was to evaluate how LF affects human the ability of ADSCs to differentiate into osteoblasts at various stimulation levels. Furthermore, we compared the in vitro osteogenic ability of ADSCs of LF with that of BMP-2 because of BMP-2′s well-documented strong osteogenic effects on stem cells [26,27]. By comparing the stimulating effect of LF on stem cell differentiation with that of BMP-2, we identified LF as a potent alternative bioactive valuable molecule for bone tissue engineering, thus promoting the application of molecules-based stem cell therapy in bone repair.

## 2. Results

### 2.1. Cell Proliferation Assay

**Figure 1:**
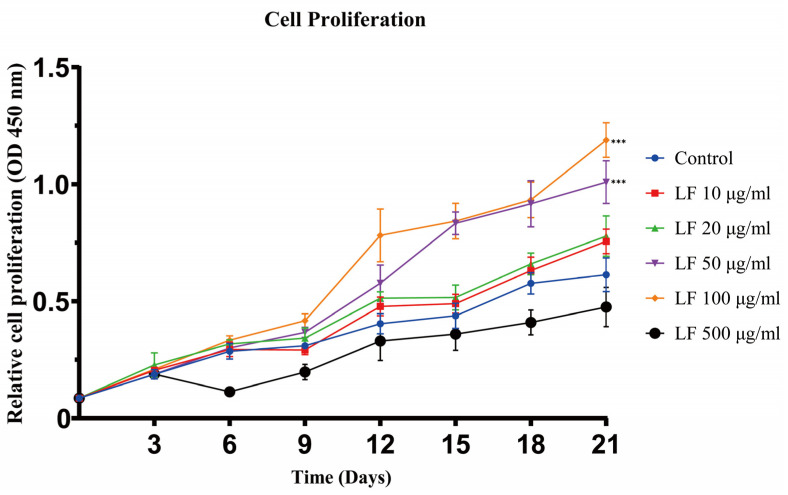
Cell proliferation assay of ADSCs cultured under different LF concentrations was analyzed on days 3, 6, 9, 12, 15, 18, and 21. LF promoted the proliferation of hADSCs at 10~100 μg/mL. Proliferation was inhibited by 500 μg/mL LF. Values are presented as means ± SE, *** *p* < 0.001.

The results of cell proliferation experiments showed that LF could promote the proliferation of ADSCs at concentrations of 10~100 μg/mL (Figure 1). From days 0 to 21, as the concentration of LF increased from 0 to 100 μg/mL, the proliferation of ADSCs accordingly increased. On day 21, the 50 and 100 μg/mL LF groups exhibited similar proliferative activity, which was significantly higher than that of the control. After day 3, 500 μg/mL LF, in contrast, harmed the proliferation of ADSCs. The experiment outcomes demonstrated that 100 μg/mL LF was the optimal concentration to encourage ADSC proliferation.

### 2.2. Alkaline Phosphatase (ALP) Staining

**Figure 2:**
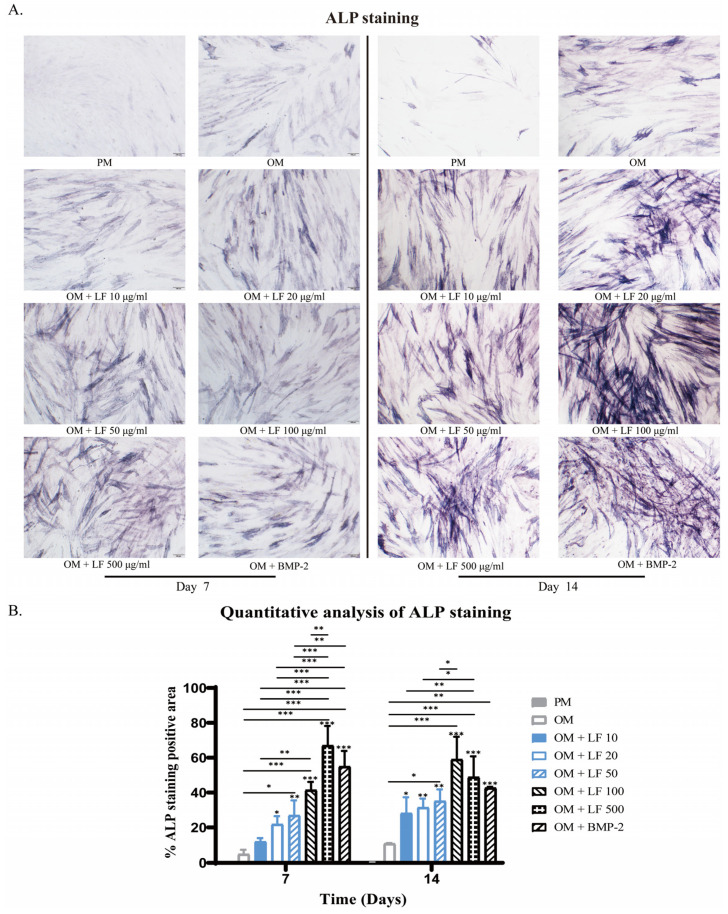
ALP staining of ADSCs on days 7 and 14 of osteogenic differentiation, photographed at 10× magnification under a light microscope. (**A**) ALP staining of hADSCs after treatment with LF or BMP-2. (**B**) Quantitative analysis of ALP staining. On the top of each column without a capped line, significance levels of seven OM groups are compared with that of PM group (* *p* < 0.05, ** *p* < 0.01, and *** *p* < 0.001).

The observed ALP-stained ADSCs in LF groups were concentration-dependent. Collectively, positively stained ADSCs increased with concentration increasing. According to the results of ALP staining, ADSCs cultured under 100 and 500 μg/mL LF produced robust osteoblastic development, which was significantly higher than that of all other groups. Comparatively, 500 μg/mL LF induced the highest ALP expression level on day 7. BMP-2 treated ADSCs presented higher ALP expression than OM and low-dose LF groups. While on day 14, the highest ALP expression level was obtained in the 100 μg/mL LF group. The ALP expression level of BMP-2 treated ADSCs was similar to that of the 10–50 μg/mL LF groups, being significantly lower than that of the 100 and 500 μg/mL LF groups (Figure 2).

### 2.3. Quantification of ALP Activities

**Figure 3:**
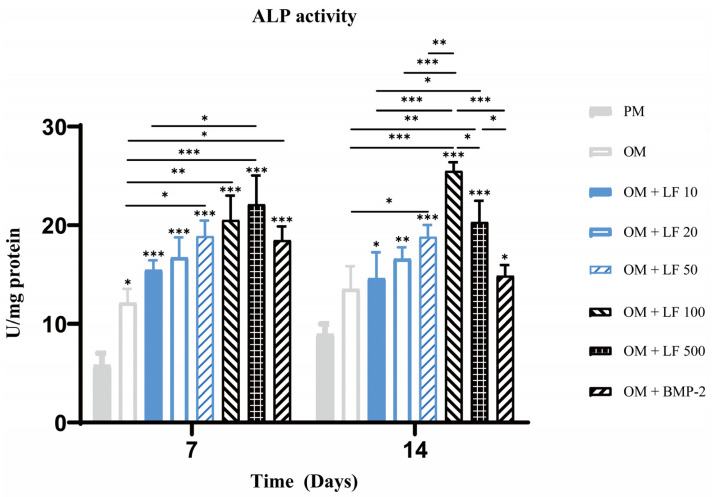
Analysis of ALP activity on days 7 and 14. On the top of a column without a capped line, seven OM groups’ significance levels are compared with that of PM group (* *p* < 0.05, ** *p* < 0.01, and *** *p* < 0.001).

ALP activity quantification experiment was also performed on days 7 and 14. On day 7, ALP activity increased with increasing LF concentration, in the range of 10 to 500 μg/mL. The 500 μg/mL LF treated ADSCs presented significantly higher ALP activity than 10 µg/mL LF treated ADSCs and OM groups. BMP-2 treatment group also showed significantly higher ALP activity than OM group. On day 14, the highest ALP activity was obtained in 100 μg/mL LF treated ADSCs. Comparatively, the ALP activity in the 500 μg/mL LF group was less than the 100 μg/mL LF group, but still higher than the other groups. The BMP-2 treated ADSCs group showed lower activity than those of the 100 and 500 μg/mL treated ADSCs groups (Figure 3).

### 2.4. Calcium Depositions of Lactoferrin and BMP-2 Induced Osteogenesis of ADSCs

**Figure 4:**
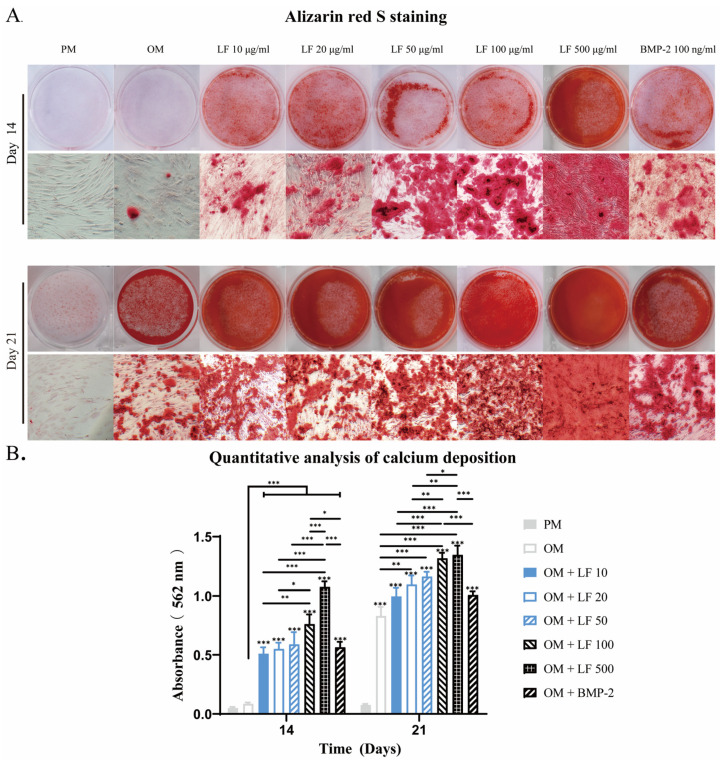
After 14 and 21 days of incubation, an analysis of calcium deposition in LF and BMP-2 treated ADSCs was conducted. (**A**) Alizarin red stains micrographs. (**B**). Semi-quantification of Alizarin red staining. Calcium was not detected in the PM group. LF and BMP-2 increased calcium deposits, and most calcium depositions were observed in ADSCs treated with LF at 100 and 500 μg/mL. On the top of a column without a capped line, seven OM groups’ significance levels are compared with those of PM group (* *p* < 0.05, ** *p* < 0.01, and *** *p* < 0.001).

Calcification is an essential process for bone formation, in which calcium phosphate accumulates within the bone matrix. Alizarin red S, with which calcium deposits are stained brilliant red, can be used for mineralizaiton detection. After 14 and 21 days of incubation, Alizarin red S mineralization analyses were conducted. Osteoblastic calcium deposition stained with brilliant alizarin red was evaluated qualitatively (Figure 4A) and quantitatively by measuring red absorption (Figure 4B).

The PM group showed no discernible calcification throughout the culture period. On days 14 and 28, the number of mineralized nodules in OM treated with LF or BMP-2 was considerably higher than in the OM group. On day 14, the LF content ranged from 0 to 500 μg/mL, and the mineralized nodules grew. The most mineralized nodules were found in the 500 μg/mL LF group. No difference in mineralized nodules was detected when comparing the BMP-2 group with the 10–50 μg/mL LF group.

At day 21, obvious mineralized nodules also appeared in the OM group, although still less than supplemented with LF and BMP-2. The 100 and 500 μg/mL LF groups exhibited the largest amounts of calcium deposits, and no statistically significant difference existed between the two groups. The comparison between BMP-2 and LF groups confirmed that the calcium deposits visibly discolored in BMP-2 treated ADSCs were similar to those in the 10–50 μg/mL LF groups but were significantly fewer than in the 100 or 500 μg/mL LF-treated ADSCs groups.

### 2.5. Gene Expression Analysis of Osteogenic Differentiation

**Figure 5:**
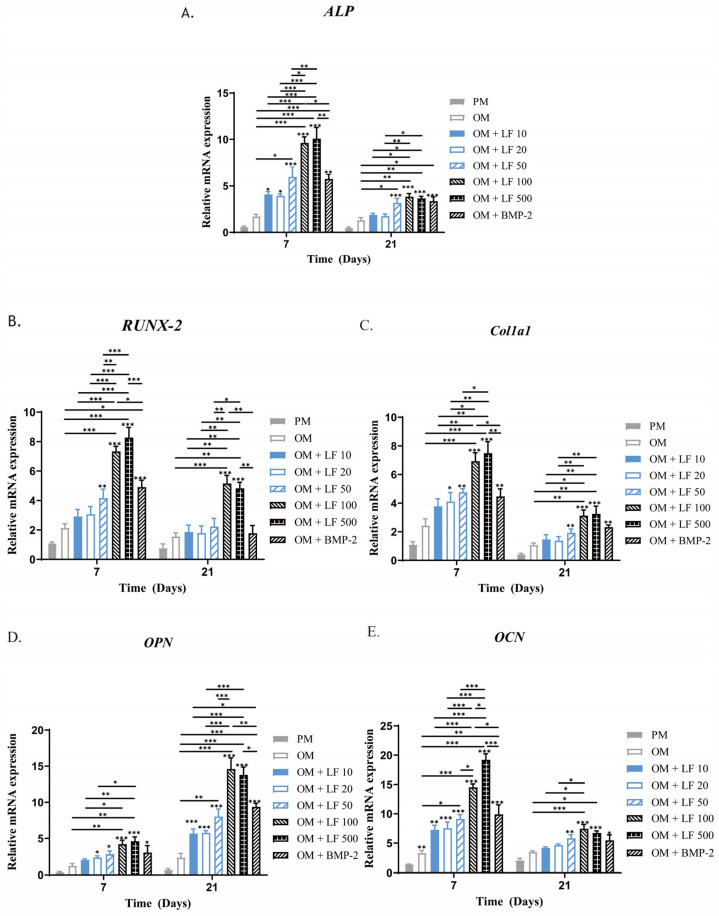
Analysis using quantitative RT-PCR of osteogenic gene expression in ADSCs exposed to LF at various doses and BMP-2 on days 7 and 21. (**A**–**E**) ALP, RUNX-2, COL1a1, OPN, and OCN expressions, respectively. All of the osteogenic marker genes (**A**–**E**) were upregulated in the LF and BMP-2 groups. Comparatively, the highest gene expression levels were stimulated by LF at 100 and 500 μg/mL. On the top of a column without a capped line, seven OM groups’ significance levels are compared with that of PM group (* *p* < 0.05, ** *p* < 0.01, and *** *p* < 0.001).

Following 7 and 21 days of incubation in an osteogenic medium supplemented with LF or BMP-2, the expression of many marker genes associated with osteogenic differentiation in ADSCs was examined using RT-PCR to determine the impact of LF and BMP-2 on osteogenesis. Most of the time, OPN, RUNX-2, ALP, COL1a1, and OCN were significantly overexpressed in all the LF and BMP-2 groups compared with in the untreated OM and PM groups, with the OM group exhibiting much higher levels than the PM group (Figure 5).

The expressions of RUNX-2 and ALP mRNA in the LF-treated groups noticeably increased with increasing LF concentration on day 7, the earliest period of cultivation, with substantially higher expressions in the 100 and 500 μg/mL LF groups than in the low-dose LF groups as well as the BMP-2 group (*p* < 0.05). The difference was not statistically significant between the 100 and 500 μg/mL LF groups. The BMP-2 group and 10–50 μg/mL LF groups did not have significantly different ALP and RUNX-2 mRNA levels. On day 21, all groups’ RUNX-2 and ALP mRNA expression levels decreased, but the overall group trends remained consistent.

Another early osteoblastic marker is COL1a1. LF treatment groups concentration-dependently increased the level of COL1a1 mRNA expression. On day 7, ADSCs incubated with 100 and 500 μg/mL LF showed significantly enhanced expression of COL1a1 compared with that of the control (*p* < 0.05). We found no significant change in the LF (10 to 50 μg/mL) group compared with the OM group. Compared with the 100 and 500 μg/mL LF groups, COL1a1 expression was considerably lower in the BMP-2 groups (*p* < 0.05). The osteogenic gene marker OPN experiences two peaks: an early peak during proliferation and the other in the later stage of differentiation. According to our findings, day 21 marked the OPN gene expression peak.

The mRNA expressions of OCN in the LF-treated groups were also enhanced with the increasing concentration. Notably, as a late osteoblastic marker, the expression level of OCN in LF-treated groups reached the peak on day 7, which is much earlier than under conventional conditions. This finding may indicate that LF can enhance osteogenesis and accelerate the process.

### 2.6. The Mechanism of LF Promoting Osteogenesis

**Figure 6:**
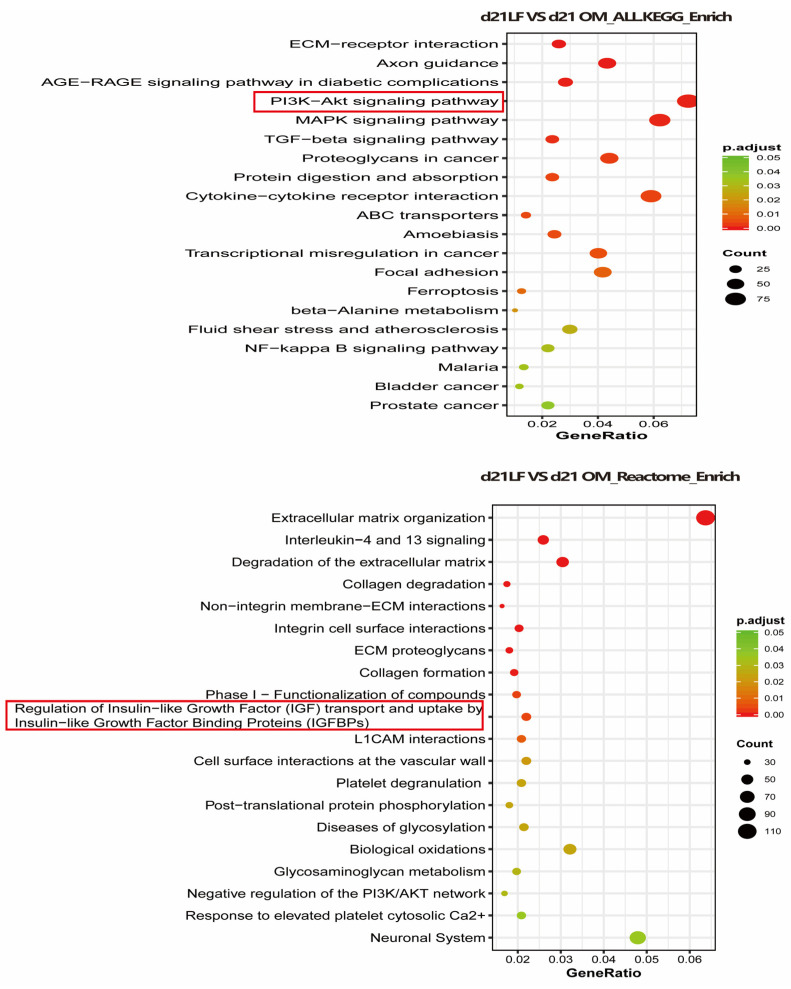
Transcriptome sequencing was performed on hADSCs of OM + LF 100 μg/mL and OM group on day 21 of osteogenic differentiation, and KEGG and Reactome enrichment of differential genes was performed. The 20 signaling pathways with the most significant changes were obtained. Red and green symbols represent up- and downregulation, respectively.

To explore the possible mechanism through which LF promotes the osteogenic differentiation of ADSCs, RNA sequencing was performed in the OM and 100 μg/mL LF-treated ADSCs groups. We used the KEGG and Reactome databases to enrich the differential genes. According to KEGG enrichment, the PI3K/AKT and MAPK pathways and the interaction between cytokines and their receptors were upregulated. Reactome enrichment indicated that extracellular matrix organization pathway up-regulation, and regulation of insulin-like growth factor (IGF) transport and uptake by insulin-like growth factor binding protein (IGFBPS) were up regulated (Figure 6).

## 3. Discussion

Since it is easy to obtain and is associated with little donor site morbidity, adipose tissue offers an interesting source of stem cells for regenerative medicine [27]. Hence, adipose-derived stem cells (ADSCs) are receiving considerable attention with respect to their osteogenic differentiation ability. Researchers have discovered several molecules for boosting the osteogenic differentiation of ADSCs, including BMP-2, BMP-6, and BMP-14, to fully exploit the osteogenic differentiation potential of ADSCs [4,28]. Since BMP-2 has been therapeutically licensed [29], and its potent osteogenic effects on stem cells have been well-reported [26,27], in this study, we used BMP-2 as a comparator stimulating differentiation of ADSCs towards the osteogenic lineage. One of the primary regulation mechanisms of the function of BMP is concentration [30]. We compared the effects of 100 ng/mL BMP-2 with those of LF because this dose apparently produces the best effect on extracellular mineralization [31].

An iron-binding glycoprotein known as LF was discovered in human and bovine milk [32]. LF has multiple bioactive functions, including immunomodulatory, anticancer, antibacterial, antiviral, and anti-inflammatory activities [32,33,34]. According to recent researches, LF may function as an anabolic factor that encourages osteoblast development and proliferation while suppressing osteoclast activity [35,36,37]. To the best of our knowledge, our study is the first to investigate the ideal dose of LF on ADSC proliferation and osteogenic differentiation and compare them with those of BMP-2.

LF levels in blood range from 2 to 7 μg/mL, although in cases of sepsis and inflammation, local concentrations can be substantially higher (1–100 μg/mL) [38]. We found that 1–100 LF μg/mL significantly and dose-dependently increased ADSC proliferation (Figure 1). Icriverzi et al. reported that LF also improved the rate of human mesenchymal stem cell (MSCs) proliferation in a similar pattern [39]. Similar to the data obtained by Liu et al. [40], increased proliferation was found in the osteoblast cell line MC3T3-E1 in the presence of LF. In a recent study, the addition of typical pluripotent mesenchymal stem cells C2C12 was also promoted by LF [41]. These consistent results demonstrate that LF in physiological concentrations (1–100 μg/mL) stimulates cell proliferation of ADSCs, osteoblasts, MSCs, and C2C12 cells. However, our study also found that a supraphysiological LF dose of 500 μg/mL inhibited the proliferation of ADSCs. Combining the effects of Alizarin red staining with this phenomenon, the mineralization process was significantly accelerated when ADSCs were exposed to 500 μg/mL LF, and apparent mineralization appeared on day 14. This could explain the higher dose of LF (500 μg/mL) may favor enhancing the differentiation rather than the proliferation of stem cells. The differentiation and proliferation of stem cells often show opposite trends [24,42].

In vivo bone formation generally undergoes three stages: initial cell proliferation, subsequent matrix deposition, and finally, matrix mineralization [43]. These biochemical events are critical for in vivo bone formation processes, which are also reflected in in vitro tissue engineering methods. Early and late differentiation of ADSCs was further evaluated by ALP staining (Figure 2), ALP activity (Figure 3), and calcium deposition (Figure 4). On days 7 and 14, ALP activity and staining in ADSCs were assessed. The maximum ALP activity occurred in the 500 μg/mL LF group on day 7, and ALP activity dose-dependently increased in the range of 10–500 μg/mL LF. However, on day 14, the ALP activity reversed in the 100 and 500 μg/mL LF groups. As shown in Figure 4, LF treatment enhanced the calcium deposition. At 500 μg/mL, LF accelerated the mineralization process of ADSCs. However, 100 and 500 μg/mL LF did not show a statistical difference in calcium deposition on day 21. The effect of 100 ng/mL BMP-2 on ALP activity and calcium deposition was significantly lower than that of 100 and 500 μg/mL LF.

To ascertain whether LF could aid ADSCs in osteogenic differentiation, the RUNX-2, ALP, OPN, COL1a1, and OCN mRNA expression levels by RT-PCR was measured. ALP, a cell-membrane-associated enzyme, is characterized as an early osteogenic marker. Increased ALP levels were generally histomorphometrically correlate with augmented bone formation. By using RT-PCR, we found that, compared with ADSCs cultivated in PM and OM and low-dose LF treatment groups (OM + LF 10, 20, and 50 μg/mL), ADSCs treated with 100 μg/mL LF (OM+ LF 100), and 500 μg/mL LF (OM+ LF 500) displayed a higher level of ALP mRNA expression. No statistically significant differences were discovered between the 100 μg/mL LF and 500 μg/mL LF groups. (Figure 5A). ALP activity correlates with matrix deposition prior to the initiation of mineralization. ALP activity and staining also significantly increased on days 7 and 14 in the LF (100 and 500 μg/mL) compared with the other groups, which was consistent with the RT-PCR results.

The transition of stem cells into the osteoblastic lineage is facilitated by RUNX-2, a transcription factor required for osteoblast development. The RUNX-2 gene expression levels in the 10, 20, and 50 μg/mL LF treatment groups were comparable. On days 7 and 21, the 100 and 500 μg/mL LF treatment groups showed noticeably higher RUNX-2 gene expression. BMP-2-treated ADSCs expressed higher RUNX-2 mRNA levels than those in the OM and PM groups, but expression was still much lower than that in the high-dose LF-treated groups (Figure 5B). The outcomes demonstrated that RUNX-2 operated as a crucial transcription factor in ADSCs following high-dose LF treatment. We obtained similar results for OPN gene expression, another nonspecific bone marker, with an early peak during the proliferative stage and another during mineralization.

COL1a1, the most abundant protein in the bone matrix, is also one of the important osteogenic markers. With increasing LF concentration, the mRNA gene expression of COL1a1 also enhanced compared with that of the OM group. However, the COL1a1 gene expression significantly improved in ADSCs cultured with LF (100 and 500 μg/mL) on days 7 and 21. The BMP-2 treated group also showed increased COL1a1 expression compared with the OM group, but still significantly lower expression than the 100 and 500 μg/mL LF-treated ADSCs (Figure 5C).

OCN appeared late and is among the most specific markers controlling matrix mineralization [44]. As shown in Figure 5E, we observed a significant increase in OCN gene expression in both the high-dose LF (100 and 500 μg/mL) and BMP-2-treated groups compared with that of the OM group, whereas 100 and 500 μg/mL LF-treated ADSCs groups showed significantly higher OCN expression than the BMP-2 group. Low-dose LF treatment increased the OCN expression of ADSCs. However, between the low-dose LF and OM groups, no discernible increase was seen. The peak of OCN gene expression in the LF treatment groups appeared on day 7 of detection. This agreed with the findings of Alizarin red staining, indicating that LF treatment could significantly accelerate osteogenic differentiation, especially in the high-dose LF groups (100 and 500 μg/mL). Of note, in the above detection of osteogenic genes’ expression, 500 μg/mL LF treated ADSCs did not show significantly increased osteogenic gene expression compared with the 100 μg/mL LF treatment group, indicating that a concentration of more than 100 μg/mL could not further improve the osteogenic performance of ADSCs.

Although LF regulates stem cell osteogenesis through multiple signaling pathways, the mechanism through which LF encourages stem cell osteogenic development is still unknown. Current research points towards the role of LF in promoting anabolic are mainly mediated by LRP1 (a functional mitogenic receptor of LF) [45]. The mitogenic mechanism through which LF enables osteoblasts also involves the activation of PI3 kinase/Akt or p42/44MAPKinase signaling pathways [46]. In addition, LF induces proliferation and inhibits apoptosis in an IGF-R1-dependent manner [47]. In our study, the PI3 kinase/Akt and IGF-R1 signaling pathways were significantly activated in LF-treated ADSCs, suggesting that the effect of LF on ADSCs may also be achieved through the signaling pathways mentioned above (Figure 6).

Considering the importance of the synergistic regulation of bioactive molecules on stem cell proliferation and differentiation, 100 μg/mL LF was superior in inducing differentiation and promoting proliferation and appeared to be the optimal concentration for bone tissue engineering. As demonstrated in previous studies, BMPs show considerable promise for regenerative medicine and have been approved for clinical application. However, compared with BMP-2, LF promoted significantly increased osteogenic differentiation of ADSCs and may avoid the high economic burdens and toxicity at high doses caused by BMP-2. As a multifunctional protein, LF also plays a role in the modulation of the inflammatory response and the immune system [48], and may be an effective therapeutic agent for bone destruction diseases associated with inflammation. LF is expected to be clinically used in combination with biomaterials as an innovative molecular and cellular therapy to promote bone repair.

## 4. Materials and Methods

### 4.1. Human ADSC Harvest

Subcutaneous adipose tissue was used to harvest human ADSCs. The medical ethical committee at ZhongNan Hospital of Wuhan University approved the study (2016001). Before being digested at 37 °C for 40 min with agitation using a collagenase A solution (0.2%, Sigma-Aldrich, Taufkirchen, Germany) in Dulbecco’s modified Eagle’s medium (DMEM, Gibco, Waltham, MA, USA), phosphate-buffered saline (PBS) with 180 IU/mL penicillin/streptomycin and 0.75 µg/mL sodium streptomycin was used to rinse adipose tissue. The fetal bovine serum (FBS; 10%, Gibco, Waltham, MA, USA) inactivated collagenase before the stromal vascular fraction (SVF) was centrifuged and pelleted. The cell pellet was then reconstituted, put through a 100 μm filter (BD Biosciences), and planted into Petri dishes. In a humid incubator at 37 °C, 5% CO_2_ primary cells were grown and kept in DMEM with 15% FBS and 1% antibiotics. Every three days, the medium was changed. In this investigation, human ADSCs were employed in passage 3.

### 4.2. Cell Culture Conditions

The osteogenic medium (DMEM supplemented with 1% antibiotics, 10% FBS, 100 nM dexamethasone, 50 μM L-ascorbic acid 2-phosphate acid, and 10 nM β-glycerophosphate) was used to culture the ADSCs, which were seeded on 24-well plates at a density of 2 × 10^4^ cells per well. The osteogenic medium also contained various concentrations of LF ranging from 10 to 500 μg/mL or 100 ng/mL BMP-2. Control groups were ADSCs that were grown in an osteogenic medium (OM) or regular proliferation media (PM). Every three days, the cultural media was replaced.

### 4.3. Cell Counting Kit-8 (CCK-8) Test for Measuring Cell Proliferation

Following the manufacturer’s recommendations, the CCK-8 cell proliferation assay was carried out in each group at 0, 3, 6, 9, 12, 15, 18, and 21 days of culture. In a nutshell, 100 μL of CCK-8 solution was used in place of the culture medium, and the incubator was incubated at 37 °C for 2 h. Then, the absorbance (450 nm) was measured using a microplate reader (Multiskan MK3, Thermo Fisher Scientific, Waltham, MA, USA). ADSCs cultured without the supplement of LF were used as control. Nine samples from each group were used, and each measurement was performed in triplicate.

### 4.4. Alizarin Red S Staining

Alizarin red S staining identified calcium deposition at the designated time points following induction. Cells were first fixed with 70% (*v*/*v*) ethyl alcohol (ETOH), followed by 10 min at room temperature with a 2% (*v*/*v*) Alizarin red (Sigma-Aldrich) staining, and then PBS washing. Under a light microscope, images were taken after staining. Alizarin red was stained for 30 min at room temperature with 10% (*v*/*v*) cetylpyridinium chloride in 10 mM sodium phosphate to measure calcium deposition quantitatively. Using a standard calcium curve in the same solution, the concentration was calculated using a multi-plate reader to determine the absorbance at 562 nm. For the quantitative analysis, each group received a minimum of 9 samples. Values were presented as the mean ± standard error.

### 4.5. Alkaline Phosphatase (ALP) Staining

The osteogenic differentiation process was stained with ALP on days 7 and 14.4% paraformaldehyde fixative was used to fix cells for the first 10 min. Cells were cleaned with PBS three times after the fixative was removed. The staining solution was withdrawn, and the sample was rinsed in pure water after 4 h of staining following the directions on the Alkaline Phosphatase Assay Kit (Beyotime, Shanghai, China). Under the microscope, pictures were taken. Image J software (Version 1.6, NIH, Bethesda, MD, USA) was used for quantitative analysis. The total area of each well and the positively ALP-stained region were determined. For each sample, values are reported as a mean percentage of the positive factor.

### 4.6. ALP Activity Determination

Assays of ALP activity were carried out on the same day as ALP staining. Physiological saline was used to wash the cells three times. and 0.1 M Tris buffer (pH 7.4) with 1% Triton X-100 was used to sonicate and scrape the cell layers. An ALP activity diagnostic kit (Beyotime) was used to detect the amount of ALP activity in the cell lysate. The Bicinchonic acid (BCA) protein analysis kit (Beyotime, Shanghai, China) was used to analyze proteins, and ALP activity was adjusted to the total protein.

### 4.7. RNA Extraction and Real-Time Polymerase Chain Reaction Analysis (RT-PCR)

Total RNA was isolated from cells using Trizol reagent (Qiagen, Hilden, Germany) after 7 and 21 days of incubation. Then, cDNA was synthesized using the manufacturer’s instructions using a Hiscrippt II Reverse 1 Strand cDNA Synthesis kit (Vazyme, Nanjing, China). Using the Light Cycler 96 thermocycler (Roche Diagnostics, Penzberg, Germany) and SYBR qPCR Master Mix (Vazyme) to detect the expressions of collagen type I alpha 1 (COL1a1), ALP, runt-related transcription factor 2 (RUNX-2), osteocalcin (OCN), osteopontin (OPN), and the housekeeping gene glyceraldehyde-3-phosphate-dehydrogenase (GAPDH) expressions. Denaturation at 95 °C for 180 s was followed by 40 cycles of 95 °C for 10 s, 60 °C for 30 s, and 72 °C for 30 s in the PCR process. Three copies of each gene analysis were carried out. The 2^−ΔΔCt^ method was used to calculate the relative gene expression. Table 1 lists the primer sequences for the selected genes.

### 4.8. RNA-Seq

On the 21st day of osteogenic differentiation, the total RNA from the OM and the 100 μg/mL LF groups were isolated using Trizol Reagent. NEBNext^®^ Poly(A) mRNA Magnetic Isolation Module kit (NEB, Ipswich, MA, USA) was used to separate the mRNA from 1 μg of RNA, and NEBNext^®^ UltraTM II mRNA Library Prep Kit (NEB, Ipswich, MA, USA) was used to create the mRNA library. The Novaseq 6000 instrument and Novaseq S4 reagent kit were used for library sequencing. Using the DESeq R program (1.18.1), differential expression analysis of two groups was carried out. DESeq offers statistical techniques for detecting differential expression in digital gene expression data using a model based on the negative binomial distribution. The *p*-values that resulted from this were modified using Benjamini and Hochberg’s method for reducing the false discovery rate. Genes identified by DESeq with |log2(FoldChange)| > 1 and an adjusted *p* value < 0.05 were classified as differentially expressed. We implemented KEGG enrichment analysis and visualized the differentially expressed genes using the cluster profile R package. Differentially expressed genes were thought to be highly enriched if the adjusted *p*-value was <0.05.

### 4.9. Statistical Analyses

For each evaluation, nine samples from each group were used, and each measurement was performed in triplicate. The data are shown as mean ± SE. GraphPad Prism 9 software (GraphPad Software Version 9.0) was used to evaluate statistical significance using an unpaired Student’s *t-*test or one-way analysis of variance (ANOVA), as necessary. Differences were deemed statistically significant when *p* < 0.05.

## 5. Conclusions

In conclusion, we have shown that LF could significantly increase ALP activity, the expression of osteogenesis-related mRNA, and the development of mineralized nodules in ADSCs. The optimal LF concentration for use as a growth factor in bone tissue engineering is 100 μg/mL. Compared with BMP-2, which is approved for clinical application, LF exhibited stronger osteoinductive efficacy, and the physiological concentration also indicates clinical safety. Our study offers a new concept for using bioactive molecules to enhance stem cell osteogenesis and presents the feasibility of using LF instead of BMP-2 for bone tissue engineering. In the future, LF is expected to be clinically used with biomaterials as an innovative molecular therapy to promote bone repair. However, the efficiency and safety of LF still need to be confirmed by long-term in vitro studies, followed by validation in vivo. Future studies should focus on determining the cellular and molecular mechanisms through which stem cells treated with LF behave in both vitro and in vivo.

## Figures and Tables

**Table 1:** Primers for real-time PCR.

Gene	Prime Sequence (5′-3′)	
GAPDH	Forward	CCT CAA GAT CAT CAG CAA
	Reverse	CCA TCC ACA GTC TTC TGG GT
COL1a1	Forward	GAG AGC ATG ACC GAT GGA T
	Reverse	ATG TTT TGG TGG TTC AGG AGG
ALP	Forward	CGC TGT GTC AAC TCC ACC T
	Reverse	CCA GAA GGT TCT GTT AAC TTG
RUNX2	Forward	GCG TCA ACA CCA TCA TTC TG
	Reverse	CAG ACC AGC AGC ACT CCA TC
OPN	Forward	TGC CAG CAA CCG AAG TTT TC
	Reverse	CTG GAT GTC AGG TCT GCG AA
OCN	Forward	GTG CAG CCT TTG TGT CCA AG
	Reverse	GTC AGC CAA CTC GTC ACA GT

## Data Availability

The data presented in this study are available on request from the corresponding author.
